# Graduate entry to medicine: widening psychological diversity

**DOI:** 10.1186/1472-6920-9-67

**Published:** 2009-11-13

**Authors:** David James, Eamonn Ferguson, David Powis, Miles Bore, Don Munro, Ian Symonds, Janet Yates

**Affiliations:** 1 Medical Education Unit, Faculty of Medicine and Health Sciences, University of Nottingham, UK; 2 School of Psychology, University of Nottingham, Nottingham, UK; 3 School of Behavioural Science and Psychology, Faculty of Science and Information Technology, University of Newcastle, Newcastle, NSW, Australia; 4 Discipline of Reproductive Medicine School of Medicine & Public Health, Faculty of Health, University of Newcastle, NSW, Australia

## Abstract

**Background:**

At Nottingham University more than 95% of entrants to the traditional 5-year medical course are school leavers. Since 2003 we have admitted graduate entrants (GEM) to a shortened (4-year) course to 'widen access to students from more disadvantaged backgrounds'. We have recently shown that the GEM course widens academic and socio-demographic diversity of the medical student population. This study explored whether GEM students also bring psychological diversity and whether this could be beneficial.

**Methods:**

We studied: a) 217 and 96 applicants to the Nottingham 5- and 4-year courses respectively, applying in the 2002-3 UCAS cycle, and, b) 246 school leavers starting the 5-year course and 39 graduate entrants to the 4-year course in October 2003. The psychological profiles of the two groups of applicants and two groups of entrants were compared using their performance in the Goldberg 'Big 5' Personality test, the Personal Qualities Assessment (PQA; measuring interpersonal traits and interpersonal values), and the Lovibond and Lovibond measure of depression, anxiety and stress. For the comparison of the Entrants we excluded the 33 school leavers and seven graduates who took the tests as Applicants.

Statistical analyses were undertaken using SPSS software (version 16.0).

**Results:**

Graduate applicants compared to school leaver applicants were significantly more conscientious, more confident, more self controlled, more communitarian in moral orientation and less anxious. Only one of these differences was preserved in the entrants with graduates being less anxious. However, the graduate entrants were significantly less empathetic and conscientious than the school leavers.

**Conclusion:**

This study has shown that school leaver and graduate entrants to medical school differ in some psychological characteristics. However, if confirmed in other studies and if they were manifest in the extreme, not all the traits brought by graduates would be desirable for someone aiming for a medical career.

## Background

There is a central policy strategy in the UK that higher education should allow greater access to students from disadvantaged backgrounds [1,2]. Historically, the medical school intake in the UK does not reflect a great demographic or academic diversity, as it comprises a far higher proportion of individuals from socio-economic group 1 and females than the population at large [3]. 'Widening access' in medical education implies an approach that supports positive discrimination, whereby more medical students with lower socio-economic status or from educationally deprived backgrounds will be selected. One practical approach to solve the under-representation of these groups has been to establish Graduate Entry to Medicine courses in UK medical schools.

This study is an investigation centred on a single UK medical school. At Nottingham more than 95% of entrants to the traditional 5-year medical course have been school leavers. Since October 2003 we have taken graduate entrants (Graduate Entry to Medicine, GEM) into a shortened (4-year) course in an attempt to 'widen access to students from more disadvantaged backgrounds'. We have shown previously that the GEM course increases the diversity of the student body with respect to age, educational achievement and socio-economic background [4]. In addition, it results in a greater proportion of male students being admitted and an ethnic profile which approaches that of the general population.

We hypothesised that GEM students increase the psychological diversity (that is, the variability in a wide variety of personality traits) as well. This study addressed two questions. First, did GEM students increase the psychological diversity of the medical school population? Second, if a difference in psychological profile was found in GEM students, was that desirable? By desirable we mean that the inclusion of the GEMs students increases the variance in personality scores, but not to extreme values. There is now evidence that high or extreme scores on 'normal' personality traits correlate with measures of abnormal personality [5-7]. Thus, increasing psychological diversity should be 'beneficial' if it leads to an overall profile where traits know to be beneficial to medical education, such as conscientiousness [3,8], show an increase but not to an extreme level.

## Methods

The research was centred on the University of Nottingham medical school which admits school leavers to its five year medical course (BMedSci; approximately 250 entrants annually) and university graduates to its four year course (GEM; approximately 90 entrants annually). Ethical approval was obtained from the Faculty of Medicine and Health Sciences Research and Ethics Committee.

Selection for the 5-year course is on the basis of academic (A-levels mainly) and non-academic criteria comprising a personal statement on the Universities and Colleges Admission Scheme (UCAS) form, a web based questionnaire and a semi-structured interview. The semi-structured interview is undertaken by two trained interviewers over 20 minutes. The interviewers are usually doctors (academic, NHS hospital or general practitioners) or medical school administrative staff, There are a series of set questions that are used to assess two candidate attributes - a) motivation and insight into a medical career, and, b) empathy. Once the interview is completed the interviewers also score the communication skills of the candidate. Criterion referenced scoring is used for all three domains.

Entry to the graduate 4-year programme is on the basis of performance in the Graduate Medical School Admissions Test (GAMSAT) examination and a structured interview for those performing well at GAMSAT (top 15% approximately). GAMSAT is a commercial test comprising three sections which are presumed to be valid and reliable [9] (see http://www.acer.edu.au/tests/university/gamsat). The 40 minute structured interview is conducted by three trained interviewers (typically a clinician, an academic and a lay person) with one acting as a chair. Each interviewer asks a different set of scripted questions aimed to discover whether the candidate a) has a realistic view of being a doctor, b) is interested medicine, and c) has personal attributes necessary for the study and practice of medicine.

The research was performed in the 2002-03 UCAS cycle and comprised two prospective studies, one on a group of applicants and one on a group of entrants.

### Applicant study

All 'home'/EU applicants to the two Nottingham medical courses were invited by letter to participate in a voluntary (Ethics Committee approved) research project. Applicants were invited to attend one of four centres (Bristol, London, Derby and Nottingham) in the Christmas holiday period (2002-3) to undertake invigilated psychological tests described below.

### Entrant study

All entrants to the two Nottingham medical courses commencing in October 2003 were invited by letter and email to participate in a voluntary (Ethics Committee approved) research project. They were invited to undertake invigilated psychological tests on a specific day in October 2003 at two sites - the Medical School building in Nottingham for the students on the 5-year course and the Medical School building in Derby for the students on the 4-year course.

A battery of psychological tests was administered to both the Applicant and Entrant participants. Medical students ideally should be emotionally stable, open minded, cooperative, trustworthy and conscientious, able to balance the needs of individuals with those of society as a whole, and who are confident and empathic. However, none of these traits should be in the extreme. Thus, we chose tests which profiled the applicants and entrants in terms of their personality, moral orientation and tendency to anxiety, depression and stress. The tests used are detailed below. All participants were provided with Information Sheets prior to the tests and provided informed signed consent.

#### The (Goldberg) Big Five

This is based on a well established and validated model of normal personality, shown to underpin many existing trait-based schemes of personality. It is a general self-report measure of personality rather than being specific to medicine. The Big Five measures in 5 domains: (1) emotional stability (emotionally reactive *vs *stable), (2) surgency (introverted *vs *extroverted), (3) intellect (open to new ideas vs. close-minded), (4) agreeableness (cooperative/trustworthy vs antagonistic) and (5) conscientiousness (hard working, well organised vs disorderly). Goldberg's 35 bi-polar markers were used in this study [10].

#### Personal Qualities Assessment (PQA)

This battery consists of three tests and was specifically developed for use in selecting medical students (see http://www.pqa.net.au) [11,12]. The first test (Mental Agility Test, MAT) is not reported here. The MAT is a measure of general cognitive ability (essentially fluid intelligence) producing a single score. It was not used in this study because it measures academic ability rather than a psychological trait. The second instrument is a measure of moral orientation (the 'LibCom' scale) [13] and produces a single score with high scores indicating a communitarian/rules-based moral orientation and low scores indicating a libertarian moral orientation. The third PQA instrument is the NACE scale [11] which measures personality traits considered to be undesirable (narcissism, aloofness) and desirable (confidence in dealing with others and empathy) in those intending a career in medicine. Based on large samples of medical school applicants and students in Australia and several other countries the Cronbach alpha reliabilities of the LibCom and NACE scales have been found typically to range between .88 and .92. Evidence of convergent and discriminant construct validity has also been reported [13,14]

#### A generalised tendency towards Depression, Anxiety and Stress

It is well-established that medical courses are stressful, and that students who are vulnerable to anxiety or depression may perform less well [15-17]. Thus, we chose to include a test with items based on the Lovibond & Lovibond (1995) Depression Anxiety Stress Scales (DAS) but reworded to reflect generalised tendencies (traits i.e., a person's typical, but not necessarily current state) rather than event specific reactions [18].

### Statistical Analysis

This was undertaken using SPSS software (version 16.0). Because of the small size of some of the groups non-parametric tests were used. Thus, Chi Square was used for comparison of frequencies and Mann Whitney U for comparison of medians.

## Results

Cronbach Alpha Reliability coefficients were calculated for all measures and are given in Table 1. All measures were found to have acceptable internal reliability.

**Table 1:** Cronbach Alpha Reliability Coefficients for Applicant and Entrant Samples

Measure	Trait	Applicant Sample n = 313	Entrant Sample n = 285	Test Retest* n = 40
Goldberg Big 5	Emotional Stability Surgency Intellect Agreeableness Conscientiousness	.76 .78 .74 .81 .77	.82 .83 .72 .83 .79	.61 .79 .53 .72 .75

PQA	LibCom: Moral Orientation	.87	.86	.50
	NACE:			
	Narcissism Aloofness Confidence Empathy	.79 .78 .82 .79	.83 .82 .82 .80	.48 .74 .84 .79

DASS	Depression Anxiety Stress	.82 .73 .77	.87 .78 .81	.72 .65 .78

* 40 participants completed the tests at application and 11 months later on entrance to their course.

As 40 participants (33 school leavers and 7 graduates) completed the tests at both application and at entrance, test/retest reliability coefficients could be calculated and these also are given in Table 1. The test/retest coefficients generally indicated acceptable temporal consistency with only moral orientation (0.50) and narcissism (0.48) having values that were lower than desirable (ie coefficients of 0.50 or less).

### Applicants

Overall, 217 of 2393 applicants (9.1%) for the 5 year course and 96 of 1235 applicants (7.8%) for the 4 year course agreed to participate in the study. The comparisons of psychological parameters in the two groups are shown in Table 2. Significant differences were found on 4 of the 13 traits measured. The applicants for the graduate program recorded significantly higher Goldberg 'Conscientiousness' scores. The graduate applicants were also more confident, more communitarian in moral values ('LibCom'), and were less anxious. No differences on the remaining 9 traits reached significance.

**Table 2:** Comparison of psychological tests in Applicants; values are medians (ranges)

Psychological Test	Criterion	'School Leavers' (5-year course) n = 217		Graduate Entry (4-year course) n = 96	
		
		Median	Range	Median	Range
Goldberg	Emotional Stability	47	18-63	48	30-58
	
	Agreeableness	54	18-63	54	37-63
	
	Intellect	52	34-63	52	40-63
	
	Surgency	48	22-61	49	22-60
	
	Conscientiousness	52	18-63	54	34-61*

PQA	Moral Orientation (LibCom)	114	86-153	120	86-156***
	
	NACE - Narcissm	52	35-72	53	28-69
		
	Aloofness	47	30-67	46	32-64
		
	Confidence	70	42-92	72	49-87**
		
	Empathy	73	60-91	72	52-89

DASS	Depression	13	9-35	12	9-29
	
	Anxiety	17	10-28	14	9-34***
	
	Stress	19	9-30	19	9-28

Key:

* p = < 0.05

** p = < 0.01

*** p = < 0.0001

### Entrants

There were 250 entrants to the 5-year course and 90 to the 4-year course in October 2003. Of these, 246 (98%) and 39 (43%) respectively took the test in the first month of their course. Of these, 33 of the entrants to the 5-year course and seven of the entrants to the 4-year course took the tests as applicants (see previous section). As a consequence therefore, we excluded these 40 students from the analysis of the Entrants to avoid double counting and bias from a within subject measurement influence embedded within a between groups analysis.

The profiles of 213 school leaver entrants and 32 graduate entry entrants are shown in Table 3. Again there were some significant differences in test scores between the two groups. However, these did not relate to all the same characteristics as the applicants, or vary in the same direction. Compared to school leaver entrants the graduate entrants again were significantly less anxious. None of the other significant differences found in the applicants, being more confident and more communitarian in oral values, were found in the graduate entrants. Indeed, in contrast to the applicant cohort, the graduate entrants had significantly lower Goldberg Conscientiousness scores. Finally, there were three new findings namely that the Graduates had lower Agreeableness but higher Aloofness scores and were less empathetic. No other differences reached statistical significance.

**Table 3:** Comparison of psychological tests in Entrants; values are medians (ranges)

Psychological Test	Criterion	'School Leavers' (5-year course) n = 213		Graduate Entry (4-year course) n = 32	
		
		Median	Range	Median	Range
Goldberg	Emotional Stability	47	15-61	46	32-58
	
	Agreeableness	53	35-63	50	36-60***
	
	Intellect	50	34-63	48	37-61
	
	Surgency	48	24-61	48	26-56
	
	Conscientiousness	51	34-63	47	33-60***

PQA	Moral Orientation (LibCom)	115	86-143	117	89-134
	
	NACE - Narcissm	53	33-76	54	43-66
		
	Aloofness	47	28-67	51	33-66*
		
	Confidence	67	45-87	68	51-80
		
	Empathy	74	58-93	71	51-86*

DASS	Depression	13	9-33	13	9-26
	
	Anxiety	18	9-31	14	9-25**
	
	Stress	21	11-33	21	13-33

Key:

* p = < 0.05

** p = < 0.01

*** p = < 0.0001

## Discussion

This study demonstrated that graduate applicants compared to school leaver applicants were significantly more conscientious, more confident, more self controlled, more communitarian in moral orientation and less anxious. Only one of these differences was preserved in the entrants with graduates being less anxious. However, the graduate entrants were significantly less empathetic and conscientious than the school leavers.

The principles of 'widening access' to higher education are accepted to be sound in principle but evidence of the value of the approach is lacking both in general [19], and specifically in respect of selecting health professionals [8] and medical students [20]. Our study was an attempt to examine whether widening access by the strategy of Graduate Entry to medicine, increased psychological diversity and that such an increase, if found, was desirable. In principle, the goal is to recruit students who can cope successfully with the demands of the course and become caring and competent doctors. In particular they should be less rather than more susceptible to depression, anxiety and stress. We would like entrants to have psychological profiles that are not at the extremes of the scale. Diversity in personality in the medical workforce is important as there is evidence that different specialty choices may be influenced by personality traits [21,22].

We acknowledge that the study has limitations, mainly related to study numbers. The sample sizes in some subgroups limited the comparisons that could be made. For example, only seven applicants to the 4 year graduate entry course and 30 to the 5 year school leaver course completed the tests at both application and entry. The ideal would have been to have sufficient numbers to allow a full 'within subjects' comparison (tested at application and entry) by course type (5 or 4 year course) and to also consider those who completed the tests at application but were not offered a place. The logistical problems of increasing the numbers especially in the applicants should not be underestimated. Increasing the number of participating medical schools may be one approach but it would introduce another confounder to the analysis and the number of students from an individual medical school may not be increased.

The key question for the interpretation of the results is how representative are the groups participating in the studies. We addressed this by undertaking subgroup analysis using the same academic and socio-demographic criteria we reported previously [4]. The school leaver applicants who participated were significantly younger (though the median age was 18 y in both groups), had a higher proportion of white individuals (67% vs 60%) and higher prior academic achievement (Median UCAS Tariff Points 450 vs 360; UCAS Tariff Points are awarded to students on the basis of their achieved A-level grades - ie A = 120 points, B = 100 points etc) than those who did not participate. Thus, the school leavers who participated were different by academic and socio-demographic criteria and so it might be speculated that they would respond differently to the psychological testing. In contrast, there were no significant differences in the academic and socio-demographic criteria between the graduate applicants who participated and those that did not and thus the argument of them being unrepresentative is less strong. It is even more difficult to make the case for the school entrants who participated being unrepresentative as 98% of the year group participated. However, the potential for bias still exists in the 39 4-year entrants as they only comprised 43% of the total. Whilst this group had the same academic and socio-demographic profile typical of all graduate entrants which we have reported previously [4] and in that respect could be considered 'representative' there may be other unidentified ways in which such a small sample could be biased and 'unrepresentative'.

In our view, the reasons for the low participation rates in the applicants is most likely to be due to the requirement of applicants to come to one of four centres in the Christmas holidays to take the test. It had no bearing on their application and those who participated did so out of goodwill. The high compliance rate amongst the school leaver entrants we believe was explained by the timetabling the tests in the first month of their course. Even though the voluntary nature of the study was stressed arguably it was too early for them to have realised they could exercise that choice. In contrast, we speculate that the experienced graduates, who also had a timetabled session for the tests, strategically chose not to take the test as they were more focussed on their studies rather than research.

Despite these concerns over participant numbers, we are of the view that the sample sizes in this study did allow a reasonably meaningful comparison between school leaver and graduate applicants. The relevance of the comparisons between school leaver and graduate entrants is lessened by the small numbers in the latter group. However, it should be noted that studies that have compared differences in personality scores in selection contexts have generally found that the same pattern of results is observed for between subjects designs (as here) and within subjects designs [23]. In fact the observed effect sizes are generally smaller for between subjects designs [23]. Similarly the test/retest coefficients generally indicated acceptable temporal consistency with level of stability at or greater than averages for similar traits reported in recent meta-analytic studies [24]. Overall, therefore, we feel can have some confidence in the validity of our findings for the differences between the two groups of applicants.

The differences between the two groups of applicants were perhaps not surprising with the graduates being more conscientious, more communitarian, more confident and less anxious - all attributes one might expect from individuals who have successfully complete a higher degree. In addition, they are consistent with the reported changes in personality with age and the fact that the graduates are older [25]. However, it should be noted that, whilst statistically significantly different, the actual differences are not great and likely to be somewhat subtle in terms of actual observable behavioural differences.

Of these psychological traits, the only one that was also more common in the graduate entrants was that they were less anxious. However, given the small numbers in the graduate entry group it is difficult to interpret the relevance of the differences between them and the school leaver entrants. Specifically the 39 graduates who participated were less agreeable, less conscientious, more aloof and less empathetic. Given that the 40 minute structured interview that the graduates undertake is, in part, designed to select individuals who are agreeable, conscientious, less aloof and more empathetic we are concerned by the potential significance of this finding. However, whilst the differences may be small and subtle in terms of meaningful behavioural differences, we need to undertake the study with larger and more representative graduate entrants to be sure of these findings.

The importance of measuring personal qualities in medical students has been debated frequently. We have suggested that the PQA might be a useful tool in medical student selection, though, to our knowledge, there have been no reports of actual use of psychological personality testing in selection in practice [1]. In contrast, there have been a number of reports that have shown a correlation with certain personality types and progress on the medical course. Manuel *et al*[19] using the 16 Personality Factor Questionnaire showed a significant correlation to clinical skills performance in 206 second year medical students. Specifically, the clinical skills score positively correlated to 'warmth' and negatively with 'abstractedness' and 'privateness'. Communication skills correlated positively with 'warmth', 'emotional stability' and 'perfectionism' and negatively with 'privateness' [19]. We surmise that 'warmth' may be somewhat equivalent to Empathy on the NACE test used here, and 'privateness' to Aloofness. We have shown that personality, and the domain of 'conscientiousness' in particular, was positively correlated to performance across the whole 5-year course in Nottingham medical students [8]. However, we also showed that this effect was strongest in the preclinical years and became less in the clinical years, and in a structural equation model there was a negative correlation between conscientiousness and clinical skill performance. The positive effect of conscientiousness has been shown by others too. In a study of 785 medical students, Lievens *et al*. demonstrated that conscientiousness significantly predicted end of year results in the preclinical years [20]. Furthermore, medical students who scored low on conscientiousness and high on gregariousness and excitement-seeking were shown to be significantly less likely to be successful in their exams [20].

We are unaware of any studies of personality and performance of graduates specifically. However, there have been several which demonstrate that personality influences the choice of career specialty in graduates. Petrides & McManus (2004) followed three large cohorts of students into their postgraduate careers and studied their choice using Holland's RIASEC (Realistic-Investigative-Artistic-Social-Enterprising-Conventional) Typology of careers [21]. Typical associations found were Surgery (realistic), Hospital Medicine (investigative), Psychiatry (Artistic) Public Health (Social), Administrative Medicine (Enterprising) and Laboratory Medicine (Conventional) [21]. Stilwell *et al*. studied nearly 4000 US medical graduates using the Myers-Briggs Type Indicator. They found that 'Feeling types' (in contrast to 'Thinking types') chose family medicine, while 'Thinking types' chose surgical specialties. Women were more likely to be 'Feeling types' and men 'Thinking types' [22].

These studies of personality and career choice suggest that we should be admitting students and graduating doctors suited to a range of careers with a variety of personality types though not extreme. In our earlier studies, we showed that graduate applicants brought greater diversity in socio-demographic (being older, more socio-economically deprived and more males), academic (lower prior academic achievement) and non-academic areas (a variety of personality differences and fewer themes in UCAS personal statements and references) [4]. In the selection process some of this diversity was retained (age, gender, socio-economic, academic and UCAS statements) but some was lost. However, that study acknowledged that the diversity which graduates brought did not result in a student population that was a mirror image of society and certainly that is not the goal of 'widening access'. In the current study also we have shown that graduate applicants brought a greater psychological diversity. The paper reports that the main elements of this diversity that were lost in the selection process were the differences in the personality traits and as a consequence a narrower spectrum of applicants was selected. If this finding is a true representation of the whole cohort, it is interesting to speculate whether this is an advantage or a disadvantage.

We are currently studying the impact of the GEM students on the course. The GEM students and the 'school leaver' group have different 'preclinical' training, but they are merged and mixed in their clinical years. The present study has shown that some of these desirable characteristics are more prevalent in the 4-year entrants than in the 5-year students.

## Conclusion

We have shown previously that admitting graduate entrants alongside school leavers increases the academic and socio-demographic diversity in a medical school [4]. This study has shown that there appear to be some differences in psychological characteristics between school leavers and graduate entrants to medical school. However, if confirmed in other studies and if they were manifest in the extreme, not all the traits brought by graduates would be desirable for someone aiming for a medical career.

## Competing interests

The authors declare that they have no competing interests.

## Authors' contributions

DJ Developed the original hypothesis and contributed to the planning of the study, coordinated the study, participated in the statistical analysis, wrote the first draft of the paper and coordinated the final version

EF Developed the original hypothesis, contributed to the planning of the study, participated in the statistical analysis and participated in the writing of the paper

DP Developed the original hypothesis, contributed to the planning of the study, and participated in the writing of the paper

MB Developed the original hypothesis, contributed to the planning of the study, participated in the statistical analysis and participated in the writing of the paper

DM Developed the original hypothesis, contributed to the planning of the study, and participated in the writing of the paper

IS Developed the original hypothesis, contributed to the planning of the study, and participated in the writing of the paper

JY Participated in the statistical analysis and writing of the paper

All authors read and approved the final manuscript

## Pre-publication history

The pre-publication history for this paper can be accessed here:

http://www.biomedcentral.com/1472-6920/9/67/prepub
